# Family eating and activity habits: a comparison of nutrition among nurses and non-health professionals in the Arab ethnic minority in Israel

**DOI:** 10.1265/ehpm.25-00220

**Published:** 2025-11-29

**Authors:** Aia Busnan, Miriam Theilla, Anat Amit Aharon

**Affiliations:** 1 Department of Nursing Science, Steyer School of Health Professions, Faculty of Medical & Health Sciences, Tel Aviv University, Tel Aviv, 69978, Israel; 2 Tel Aviv-Yaffo Academic College, School for Nursing Sciences, Israel

**Keywords:** Food literacy, Self-assessed nutrition, Family eating and activity habits, Arab ethnic minority, Nurse

## Abstract

**Background:**

Obesity and diabetes constitute significant health concerns within the Arab population in Israel. The study examines food literacy and self-assessed nutritional variables, which may explain family eating and activity habits among the Arab ethnic minority in Israel. The study examines these variables among professional nurses, who are expected to advocate for healthy behaviors within the population, compared to non-health professionals.

**Methods:**

A cross-sectional study compared two groups: professional nurses and non-health professionals. A closed, structured self-report questionnaire (with five sub-sections) assessed food literacy, self-assessed nutrition, and family eating and activity habits. Data was collected between January and May 2022. A MANCOVA was used to compare the research variables among nurses and non-health professionals, and η^2^ was calculated as the effect size. A multiple linear regression was conducted to examine the variables explaining families’ eating and activity habits. The model’s significance and variance explained (R^2^) were calculated.

**Results:**

The study included 213 Israeli Arab participants (103 nurses and 110 non-health professionals). Nurses showed poorer self-assessed nutrition and family eating/activity habits than non-health professionals. Regression analysis identified profession (β = −0.39, *p* < 0.001), gender (β = 0.22, *p* < 0.001), BMI (β = −0.22, *p* < 0.001), food literacy (β = 0.20 *p* < 0.001), and self-assessed nutrition (β = 0.30, *p* < 0.001) as significant predictors of family eating and activity habits. The model was significant (*p* < 0.001) and explained 38.0% of the variance.

**Conclusion:**

Despite their health education background, Arab nurses reported suboptimal nutritional behaviors and unhealthy lifestyles that impact their family lifestyle practices, potentially hindering their health and limiting their effectiveness as health role models. Policymakers should develop ongoing nutrition health promotion programs tailored to the Arab nurses and Arab ethnic minority communities in Israel.

## Background

Adopting a healthy lifestyle has been associated with low morbidity and mortality worldwide. A healthy lifestyle includes nutrition control, smoking avoidance, physical activity, controlled alcohol consumption, and controlled body mass index (BMI) [1]. These elements are considered behavioral factors promoting human health across age, gender, and culture. Adhering to healthy lifestyle principles has implications for personal health and well-being, reducing the risk of developing chronic diseases such as diabetes and cardiovascular diseases, preserving functional independence in older age, and improving quality of life [2]. Despite this evidence, research has found disparities in lifestyle behaviors across different population groups, including healthcare professionals [1]. Studies found that healthcare professionals do not adhere to healthy lifestyle recommendations, including nutrition control and weekly activity, which they maintain even less than the general population [3]. A study in Israel found that 57% of healthcare professionals suffer from obesity, 71% do not exercise regularly, and 79% do not adhere to nutrition recommendations [4]. A study among physicians in the US found that only 46% of participants were familiar with the American Heart Association recommended moderate physical activity per week (150 minutes), and only 31% adhered to the recommendation. Additionally, only 25% correctly knew the fruit and vegetable recommendations [5].

Multiple factors contribute to healthcare professionals’ non-adherence to health recommendations. These include time constraints, shift work leading to disrupted sleep patterns, and the cumulative effects of high workload and fatigue. Additionally, stress factors such as extended working hours, the responsibility of caring for complex patients, and an unsupportive or frequently changing work environment can negatively impact nurses’ and healthcare professionals’ ability to adopt healthy lifestyle practices [6, 7]. The literature suggests that despite the theoretical professional knowledge nurses and healthcare professionals possess regarding healthy lifestyles, there is a gap between knowledge and behavioral application.

Food literacy was associated with healthy lifestyle behavior and health outcomes [8]. Although there is no single definition for food literacy, the most comprehensive definition was presented by [9]:

Food literacy is the ability of an individual to understand food in a way that they develop a positive relationship with it, including food skills and practices across the lifespan in order to navigate, engage, and participate within a complex food system. It is the ability to make decisions to support the achievement of personal health and a sustainable food system considering environmental, social, economic, cultural, and political components. (p. 143)

This definition reinforces the link between food literacy and health literacy. In other words, food literacy encompasses a broad spectrum of knowledge and skills related to reading and understanding food labels, selecting food that meets the needs of individuals and their families, obtaining information about nutrition and healthy eating, healthily preparing food, and critically analyzing the relationship between food consumption and its impact on individual and community health [8]. Studies found that higher awareness and knowledge regarding nutrition were associated with increased consumption of fruits and vegetables and less consumption of fatty food. It was concluded that food literacy is essential for healthy food choices and for improving individual nutritional well-being and health [10]. There is a gap in the literature regarding the impact of food literacy on nurses’ and healthcare workers’ nutrition behavior.

Self-assessment of nutrition is a low-cost, practical, and easy-to-use tool for identifying populations at risk of nutritional disorders and alterations in nutritional status [11] and a predictor of clinical laboratory data and morbidity [12]. Self-assessment of nutrition derives from the broader concept of self-rated health. It is a subjective assessment that refers to an individual’s feelings and perceptions about their health status, as opposed to objective health status, which is defined by professional and accepted measurable parameters [13]. Self-assessed or self-rated health was found to be a good predictor of health outcomes such as chronic morbidity and mortality [14]. Regarding self-reported weight and height, studies found a good correlation between subjective reported and objective anthropometric parameters, although as age increases individuals tend to report greater height. In contrast, females tend to report lower weight than they actually are. Self-reported data may be influenced by factors such as ethnicity, culture, race, and education level [15]. Nevertheless, self-reported data are widely used for health assessment due to their ease of implementation in health monitoring systems and for assessing changes in health status [13].

### The Arab ethnic minority in Israel

Israel’s demographic includes a variety of ethnicities and religions, with the most salient being Jewish (73.9%) and Arab (21.0%) [16]. Diseases associated with a healthy lifestyle, such as obesity and diabetes, are a growing threat to Western society, including Israel, and particularly among the Arab ethnic minority in Israel. In general, Israel’s Arab ethnic minority is characterized by a lower socio-economic status compared to the majority population [17]. Worldwide, populations with lower socioeconomic status and minority groups tend to experience higher rates of morbidity and mortality [18]. Notably, over the past decade, the education level among the Arab ethnic minority in Israel has increased by 122%. Currently, approximately 17% of academic students in Israel are from the Arab ethnic minority [16]. Strong evidence exists of the association between education, food literacy, and health-related nutrition behaviors [19]. Despite the increase in education among the Arab ethnic minority, there is still a gap in knowledge regarding healthy lifestyles, including nutrition and exercise. These disparities may contribute to the increase in chronic diseases such as diabetes and cardiovascular diseases within this population [20].

Further to the literature, the aims of the current study were: a) to compare the food literacy, self-assessed nutrition, and family eating and activity habits of professional nurses and non-health professionals among the Arab ethnic minority in Israel; b) to examine variables that may explain family eating and activity habits among the Arab ethnic minority in Israel.

## Methods

### Study setting and sampling

A cross-sectional study utilizing a convenience sample.

The study was conducted among Israel’s Arab ethnic minority, focusing on two groups. One group consisted of nurses from a large hospital in central Israel working in internal care and surgical departments. All had an academic degree. The second group was non-health professionals (teachers, lawyers, high-tech professionals, and social workers). The latter were recruited from Israel’s central and northern areas using the snowball method. Inclusion criteria were adult Hebrew speakers with an academic level of education.

The sample size was calculated using G*Power software ver. 3.1.9.7 [21]. For hierarchical regression with 15 explanatory variables, power = 85%, α = 0.05, and effect size 0.15 (medium) yielded 153 participants. Considering the chance that participants would withdraw or improperly complete the questionnaire, a decision was made to include 20% more participants, such that the minimum sample size was 184. The final sample consisted of 213 participants, with a post hoc power analysis of 0.96.

### Materials

A closed structured self-report questionnaire with four sections was used, as follows:

*Personal information.* Including gender, age, marital status, average family income, number of children, years of education, height and weight, and profession (nursing/non-health professionals).

*Short food literacy questionnaire (SFLQ).* This questionnaire was developed by [22] to measure personal skills of functional, interactive, and critical health literacy regarding food and nutrition consumption. The questionnaire included 12 items, and the participants were asked to score their level of understanding regarding the nutritional values of food products, the sources of information they use regarding their nutrition, their knowledge of the Ministry of Health’s dietary guidelines, and others. The questionnaire was adapted to Israeli culture, for example, “How familiar are you with the official Israeli food pyramid?” or “I know the official Israeli Ministry of Health recommendations for salt intake”. The answers were provided on a Likert scale from 0–4 or 0–5, and the final score ranged from 7–52. The higher the score, the higher the food literacy. The original internal consistency Cronbach’s alpha was 0.82, and in the current study 0.86. The questionnaire was translated into Hebrew and back to English [23].

*Self-assessment nutrition score (SANS).* The SANS was developed by [24] in Hebrew. The SANS generates a subjective assessment of the individual’s nutrition status in the last three months. The questionnaire included ten items regarding general nutrition perception, experience of eating, evaluation of weight changes, water intake, and others. Participants were asked to score their self-assessment of each item on a Likert scale of 1–9. Mean and SD were calculated, where the higher the score, the more positive the nutrition self-assessment. A sample item is: “What is your general nutrition status today compared to the last three months?” Replies ranged from 1 = much worse to 9 = much better. The original internal consistency Cronbach’s alpha was 0.81, and in the current study 0.88.

*Family eating and activity habits questionnaire (FEAHQ).* [25] developed a questionnaire (in Hebrew) to assess eating and activity habits among families and examine family factors as risk factors for obesity. The questionnaire is suitable for assessing the eating and activity habits of the family as a single unit, as well as among individuals within the family. For the current study, only the individual assessment was used, with permission from the author. The original internal consistency Cronbach’s alpha of the full questionnaire was 0.84, and in the current study 0.80.

This questionnaire includes five dimensions as follows:

• Exercise activity. Two open-ended questions asked participants about the weekly average time they spend in exercise activities (walking, bicycle riding, or physical social action). The sum of the responses was calculated, where a higher score indicated higher health habits. A medium correlation was found between the two items (r = 0.40, *p* < 0.001).
• Family eating habits. Four items examined family eating habits. For example, “How often do you have dinner with your family?”. Replies were on a 0–4 scale, where 0 = never, 1 = rarely, 2 = occasionally, 3 = often, and 4 = always. The sum of the responses was calculated, resulting in a score ranging from 0–16. A higher score indicated healthier family eating habits.
• Screen watching. Two open-ended questions asked the participants about the average time they spend every day watching screens (computer, cellular phone, tablet). The sum of the responses was calculated, where a higher score indicated higher screen watching (non-healthy habit). The current study found a medium correlation between the two items (r = 0.31, *p* < 0.001).
• Nonhealthy eating. Five items examined nonhealthy personal eating, for example, “How often do you eat fast food every week?”. Replies were given on a 0–4 scale of 0 = never, 1 = rarely, 2 = occasionally, 3 = often, and 4 = every day. The sum of the responses was calculated, producing scores ranging from 0–20. A higher score indicated greater unhealthy eating habits.
• Poor personal eating habits. Nine items examined personal undesirable eating habits. For example, one question was, “How often do you eat directly from the pot?”, with replies on a 0–4 scale of 0 = never, 1 = rarely, 2 = occasionally, 3 = often, and 4 = always. The score calculated ranged from 0–36. A higher score indicated higher poor personal eating habits.

### Data collection

Questionnaires were distributed in two ways: hospital nurses received the questionnaire from the first author, and the completed forms were collected 24 hours later. One hundred and ten questionnaires were distributed to this group, and 103 were completed for a 93.6% response rate. The non-health professionals group was recruited by key persons who distributed questionnaires to their acquaintances of the same profession following the inclusion criteria. The key persons collected the completed forms. One hundred and twenty questionnaires were distributed to this group, and 110 were completed for a 91.6% response rate. Data was collected between January and May 2022.

### Data analysis

Descriptive statistics were used to describe the sample. A chi-square test for categorical variables and a t-test for continuous variables were used to compare between the groups. BMI was calculated by the weight and height of each participant. Scores in the SFLQ, SANS, and FEAHQ (the dependent variable) were standardized with the z-score. Skewness and kurtosis were examined, indicating normal distribution. By controlling for the socioeconomic variables, a MANCOVA was used to compare the research variables between nurses and non-health professionals, and η^2^ was calculated for each variable as the effect size. Normal probability plots were used to check the assumption of homoscedasticity. Independent errors were matched with the Dubrin-Watson test and were found to have a value of 2.14. These parameters suggested a normal distribution. A multiple linear regression was conducted to examine the explanatory variables of the dependent variable (FEAHQ). The model’s significance and variance explained (R2) were calculated. SPSS package ver. 27 was used.

### Ethical considerations

The hospital Helsinki and the university ethics committee approved the study (#8736-21-SMC and #0004530-1, respectively). Participants signed an informed consent form. Anonymity was promised, and participants were assured that the research data would only be used for statistical analysis. Participants could withdraw from completing the questionnaire at any time.

## Results

The sample characteristics were compared between the nurse and non-health professionals groups, as shown in Table 1. The nurse group was significantly younger, with fewer children and more years of education than the non-health professionals group (31.5 vs. 42.8, *p* < 0.001; 1.8 vs. 3.3, *p* < 0.001; 18.3 vs. 17.2, *p* < 0.001, respectively). The nurses had a significantly lower BMI than non-health professionals (23.84 vs. 25.3, *p* = 0.006), but both groups were within the recommended BMI.

**Table 1:** Sample characteristics comparing nurses and non-health professionals

**Variable**	**Entire** **sample** **(n = 213)**	**Nurses** **(n = 103)**	**Non-health** **professionals** **(n = 110)**	**χ^2^**	***p* =**

	**n (%)**	**n (%)**	**n (%)**		
Gender				4.53	<0.001
Male	73 (34.3)	51 (49.5)	22 (20.0)		
Female	140 (65.7)	52 (50.5)	88 (80.0)		
Religion				3.01	0.002
Muslims	190 (89.2)	85 (82.5)	105 (95.5)		
Christian	19 (8.9)	15 (14.6)	4 (3.6)		
Druze	4 (1.9)	3 (2.9)	1 (0.9)		
Marital status				7.38	<0.001
Married	127 (59.6)	35 (34.0)	92 (83.6)		
Non-married	81 (38.1)	65 (63.1)	16 (14.5)		
Other	5 (2.3)	3 (2.9)	2 (1.8)		
Children				10.07	<0.001
Yes	109 (51.2)	16 (15.5)	93 (84.5)		
No	104 (48.8)	87 (84.5)	17 (15.5)		
Income				36.13	<0.001
Below average	96 (45.1)	68 (66.0)	28 (25.4)		
Average	76 (35.7)	25 (24.3)	51 (46.4)		
Above average	41 (19.3)	10 (9.7)	31 (28.2)		

	**M (SD)**	**M (SD)**	**M (SD)**	**t**	***p =***

Age	37.3 (9.16)	31.5 (5.31)	42.8 (8.65)	11.55	<0.001
Number of children (n = 108)	3.1 (1.19)	1.8 (0.83)	3.3 (1.1)	5.14	<0.001
Education (years)	17.7 (2.07)	18.3 (2.44)	17.2 (1.47)	−3.76	<0.001
BMI	24.6 (3.90)	23.84 (3.85)	25.3 (3.83)	2.80	0.006

A point-biserial correlation was run to determine the relationship between the socioeconomic variables. There was a statistically significant positive high correlation between age and marital status (r_pb_ = 0.51, *p* < 0.001), between marital status and being parents (r_pb_ = 0.78, *p* < 0.001), between age and being parents (r_pb_ = 0.64, *p* < 0.001), and between age and group (nurses and non-health professionals) (r_pb_ = 0.60, *p* < 0.001). This means that using the “group” variable represents the participant’s age, marital status, and being a parent. Therefore, it was decided not to include age, marital status, and having children in the MANCOVA and regression model to avoid multicollinearity.

A MANCOVA was conducted to examine whether the variables consisting of the SFLQ, SANS, and FEAHQ scores (through the five dimensions) differed for nurses and non-health professionals after controlling for gender, income, and BMI. The nursing profession group showed a lower subjective nutritional assessment (SANS) score, with a small effect size (2.26 vs. 5.60, *p* = 0.02, η^2^ = 0.023) and lower family eating and activity habits (FEAHQ) with a large effect size (−4.11 vs. 3.85, *p* < 0.001, η^2^ = 0.144) than the non-health professionals (Table 2). As shown in Table 2, with the exception of exercise activity, on all parameters of the FEAHQ, nurse professionals obtained statistically significantly lower scores than the non-health professionals. There were no statistically significant differences between the groups in the food literacy (SFLQ) variable.

**Table 2:** Multivariate analysis of covariance (MANCOVA) comparing nurses and non-health professionals

**Variable**	**Nurse** **professionals**	**Non-health** **professionals**	**F**	***p* =**	**η^2^**

	**M (SD)**	**M (SD)**			
SFLQ	36.13 (6.42)	37.11 (7.46)	0.46	0.49	0.002
SANS	5.26 (1.23)	5.60 (1.08)	4.93	**0.02**	0.023
FEAHQ					
Exercise activity (hours/week)	3.92 (3.74)	2.93 (3.68)	0.11	0.73	0.001
Family eating habits	6.13 (3.30)	10.94 (3.54)	49.75	**<0.001**	0.193
Screen watching (hours/day)	5.76 (4.33)	4.75 (2.68)	4.33	**0.03**	0.020
Nonhealthy eating	10.06 (4.25)	8.40 (4.23)	13.42	**<0.001**	0.061
Poor personal eating habits	15.47 (6.63)	12.43 (5.89)	14.54	**<0.001**	0.065
Standardized FEAHQ (entire questionnaire)	−4.11 (10.67)	3.85 (9.60)	34.89	**<0.001**	0.144

SFLQ = Short food literacy questionnaire; SANS = Self-assessment nutrition score; FEAHQ = Family eating and activity habits questionnaire.

The multivariate linear regression revealed that non-health professionals (β = −0.39, *p* < 0.001), being men (β = 0.22, *p* < 0.001), having a lower BMI (β = −0.22, *p* < 0.001), higher food literacy (β = 0.20 *p* < 0.001), and higher self-assessed nutrition (β = 0.30, *p* < 0.001) may significantly explain family eating and activity habits (FEAHQ). The model was significant (*p* < 0.001) and explained 38.0% of the variance (R^2^) (Table 3).

**Table 3:** Multivariate linear regression to explain family eating and activity habits

**Variable**	**β**	**SE**	***p* =**
Professional group	−0.39	1.45	<0.001
Gender	0.22	1.41	<0.001
Income	0.07	1.33	0.28
BMI	−0.22	0.16	<0.001
SFLQ	0.20	0.10	<0.001
SANS	0.30	0.56	<0.001

Adj. R^2^ = .38, F(6, 206) = 22.69, *p* < 0.001

SFLQ = Short food literacy questionnaire; SANS = Self-assessment nutrition score. Professional group: 1 = nurse, 0 = non-health; gender 1 = male, 0 = female; income 1 = below average, 0 = average and above average.

## Discussion

The current study focused on family eating and activity habits among the Arab ethnic minority in Israel, comparing nurses and non-health professionals. The main finding is that the nurse group showed significantly lower self-assessed nutrition and family eating habits and higher screen watching, unhealthy eating, and poor personal eating habits, compared to the non-health professional group. Moreover, the nurse group was associated with worse family eating and activity habits than the non-health professional group. These findings reveal a paradoxical situation, since nurses have extensive health knowledge and a professional role as health promoters. This finding corroborates previous studies that found discrepancies in nutrition knowledge and healthy behavior among nurses and physicians, despite being health professionals [4, 26]. Other studies found that health professionals were unfamiliar with the latest dietary recommendations [5].

Nurses perceive their role as health promoters [27]. Hence, they must have up-to-date, evidence-based professional knowledge. Unfamiliarity with the latest nutritional recommendations may impair their ability to counsel their patients on this issue and consequently be detrimental to the quality of care provided [28]. Aggarwal et al. (2020) identified a mismatch between nutrition and lifestyle beliefs and actions among physicians, suggesting that healthcare professionals often struggle to practice the healthy behaviors they promote. Similarly, Power et al. (2017) discussed perceived barriers that prevent nurses from engaging in healthier eating and physical activity, emphasizing time constraints and stress as significant factors.

In a systematic review, Borges et al. (2024) found a positive correlation between healthcare workers’ (including nurses’) personal activity and healthy lifestyle counseling for their patients. These findings highlight the critical link between healthcare professionals’ personal health behaviors and their competence to counsel patients effectively. Studies emphasize the environmental health setting as a barrier to adopting healthy nutrition and lifestyle among nurses and healthcare workers [29]. A systematic review by Marko et al. [6] identified barriers to healthy eating among hospital nurses, including low accessibility and validity of healthy foods, work culture and social norms, workload and shift patterns, stress, and fatigue. A qualitative study conducted among nurses working in hospitals has shown that night shift work may disrupt circadian rhythm, which influences meal timing, food choices, and alertness. During leisure hours, habits are affected by the process of recovering from circadian disruption, often leading to fatigue, irregular meals, cravings for high-sugar foods, and reduced physical activity [30]. Another study found a significant association between the cumulative number of night shifts and higher BMI among nurses [31]. Work exigencies and hospital constraints, as well as the shortage of nurses and working night shifts, have an impact on short meal breaks, eating unhealthy convenience food, and less exercise [32]. The conclusion is that health policymakers must facilitate working conditions that will encourage a healthy lifestyle among nurses and healthcare workers. Addressing these barriers could enhance nurses’ adherence to healthier lifestyles, ultimately improving the quality of nutrition and lifestyle counseling they provide.

The current study found that nurses exhibited more unhealthy behaviors, such as increased screen time and poor personal eating patterns, compared to non-health professionals. In the current study, screen time focused on activities outside work hours. Participants may not have differentiated between work-related and leisure-related screen time, although emphasized in the questionnaire. Accordingly, the screen time reported by nurses was significantly higher than that reported by participants in the non-professional group. Working with digital medical files and needing evidence-based, up-to-date nursing and medical information as a decision-making support exposes nurses and health workers to high screen time, particularly via smartphones and computers [33]. In the last decade, the use of technology in telenursing by nurses and healthcare professionals has expanded, increasing their screen time [34, 35]. Studies have shown that high screen time was associated with the development of chronic diseases such as obesity, diabetes, depression, sleep disorders, vision disorders, and cardiometabolic diseases [36, 37]. This finding is a “call for action”, where nurses and health professionals may be a high-risk population for non-communicable diseases.

Previous studies have found that nurses exhibit eating patterns that differ from the general population, often in undesirable ways [38]. Poor dietary habits among nurses may negatively influence their families’ eating behaviors. In the current study, the nurse group showed worse family eating and activity habits compared to the non-health professional group. Moreover, being a nurse was associated with deteriorating family eating and activity habits. Research shows that family meals are a central place for family members to gather and promote healthy eating habits. The frequency of family meals is associated with healthy, high-quality eating, particularly the consumption of more vegetables and fruits and less sugar-sweetened foods and beverages [39]. Studies reinforce the role of family eating habits in shaping children’s nutritional habits and highlight the importance of targeted interventions in early childhood to prevent obesity and the critical role of family and cultural influences on family eating behaviors [25]. Having regular family meals is associated with multiple health benefits for adolescents and young adults, including healthful dietary intake, lower levels of unhealthy weight-control behaviors, and better psychosocial well-being. Intergenerational transmission of family meal routines is critical to the young generation [40].

In light of the findings of the current study and the literature on the topic, it is evident that the unbalanced dietary habits of nurses, compared to the non-health professional group, increase the risk of morbidity for their family, both in terms of nutrition and in the long-term development of obesity, diabetes, and other diseases. These findings are particularly concerning within the Arab minority in Israel, where socioeconomic disparities further exacerbate health risks [17]. Recent studies demonstrate a direct correlation between low socioeconomic status and increased consumption of processed foods [41], as well as increased prevalence of lifestyle-related diseases among the Arab ethnic minority [42]. Given these factors, there may be a heightened risk of non-communicable diseases among nurses’ families in the Arab minority. Nurses serve as agents of health behavior change within their communities; however, their suboptimal dietary habits may compromise their ability to effectively fulfill this role [43].

The current study found a significantly lower self-assessment nutrition score (SANS) among the nurse group than the non-health professionals group. Furthermore, a lower SANS was significantly associated with lower family eating and activity habits. The SANS assesses dietary patterns, weight trends, and self-perceived health relative to peers, providing a holistic view of nutritional self-awareness [24]. A study conducted in Israel found that Arab patients systematically rated their subjective nutritional status lower than did Jewish patients [44]. The current study emphasized that there are differences in self-assessed nutrition between nurses and non-health professionals from the same culture.

The dietary habits of nurses and physicians often reflect the demanding and irregular nature of their professional responsibilities [30]. These habits can significantly impact self-rated nutrition scores, particularly regarding meal regularity, nutritional quality [31], less regular breakfasts, and higher alcohol consumption [45]. Additionally, the intergenerational transmission of health behaviors is well-documented, with children frequently adopting their parents’ dietary and activity patterns [46]. Consequently, if nurses maintain suboptimal eating habits and low physical activity levels, these behaviors could negatively affect their families’ health and well-being [47]. This highlights the need for interventions that address not only the personal health behaviors of nurses but also the broader familial context [48].

Finally, the current study found no significant differences in food health literacy between the groups. However, low food health literacy was significantly associated with lower family eating and activity habits. Adults with high food health literacy were associated with increased consumption of fruits and vegetables, reduced consumption of sugary drinks and processed foods, and higher adherence to nutritional guidelines. They also frequently use nutrition labels and healthy food choices, improving overall health [49, 50]. The conclusion is that even if nurses are expected to have higher food health literacy than the public, knowledge alone does not guarantee a better health outcome for them and their families. This echoes previous observations that health knowledge does not necessarily translate into one’s own healthy behavior [51]. It can be assumed that additional factors, such as working conditions, nurses’ complex work environment, and deficient self-care [30, 32], also influence the health behavior of nurses.

The current study was conducted among the Arab ethnic minority living in Israel. Cultural influences emerged as an essential context for interpreting the study’s results. The Arab minority in Israel has distinct dietary traditions, social norms, and lifestyles that can impact nutrition and activity behaviors. Diets in the Arab community in Israel have been shifting in recent decades; a nutrition transition was observed whereby healthier traditional elements (e.g., vegetables and legumes) are being partly supplanted by processed and convenience foods. Studies in Israel found that students in the Arab sector consumed less healthy foods and had poorer diet quality compared to those in the Jewish sector [52]. Cultural norms also influence physical activity, especially for women. Arab women may face social constraints to exercising openly to avoid being seen, reflecting traditional expectations around women’s public behavior [52]. A literature review that examined 34 works worldwide found that women experience cultural, social, and religious barriers to physical activity across countries and cultures [53]. In Arab society, the nursing profession is perceived as a respectable profession that requires a higher education and ensures a secure and stable source of livelihood [54]. For Arab women, the nursing profession is perceived as an opportunity to deal with inequality and a platform for social mobility [55]. However, the role of women in Arab culture exists within the conservative framework of a patriarchal society. Women are responsible for most of the housework, raising children, and taking care of the home, regardless of whether they work outside the home [56]. Arab female nurses, who often work shifts, including weekends, experience it more intensely. Therefore, as demonstrated in the current study, these professional-cultural factors may negatively affect their ability to maintain a healthy lifestyle and a healthy diet for themselves and their families.

The cultural context in the current study reinforces the findings that nurses who are part of the Arab ethnic minority struggle with cultural barriers and norms and, at the same time, are at a higher risk of not meeting the recommended nutritional (or physical activity) goals for themselves and their families. Overall, the key findings emphasize a need to bridge the gap between knowledge and practice among nurses, address cultural and environmental barriers, the hospital work environment, and stress that even health professionals require support to maintain healthy habits. As Singh et al. [57] noted, social determinants of health play a crucial role in shaping health behaviors and outcomes, further supporting our results.

### Strengths and limitations

The primary strength of this study lies in its identification of lifestyle disparities among the Arab minority in Israel through a comparison of nurses with professionals from other academic fields. The findings highlight existing gaps within Arab society and underscore nurses as a particularly vulnerable group in terms of health risks. Furthermore, the study explored how the lifestyle practices of both groups influence their dietary habits and subsequently impact the health of their families. Thus, the results emphasize that nurses represent a high-risk group due to their unhealthy lifestyle patterns, which extend significant health risks to their family members as well. To the best of our knowledge, this research represents the first examination of this topic among the Arab minority population. Also, this is the first study to examine the issue of family eating and activity habits among Arab nurses.

The current study has several limitations. First, the nurse participants were recruited from a single medical center in Israel. Although this center is the largest hospital in Israel and includes nurses in various departments who earned their undergraduate nursing education at diverse institutions, the specific organizational culture of the medical center may have influenced the nurses’ nutrition patterns. Second, the comparison group of non-health professionals was obtained through convenience sampling from two regions in Israel, which may limit their representativeness of the broader Arab minority population. Therefore, the sampling method could impair the generalizability of the research findings. Moreover, the study did not include other minority subgroups residing in Israel, such as Christian Arabs, who have distinct cultural characteristics; their absence may further affect the generalizability of the findings. Therefore, future research should aim to include a representative sample encompassing the diversity of all minority populations in Israel. Another limitation is the high proportion of women in the non-professional group compared to the nurse group. This imbalance may have potential implications for interpreting the findings, particularly in the sociocultural context of Arab families. Nevertheless, an interaction analysis revealed that the effect of profession on family eating and activity habits did not differ significantly by gender. Finally, the study used a self-report questionnaire that may reflect personal perceptions rather than objective measures, which could influence the consistency of the findings. This methodological limitation could further impact the validity of the results.

## Conclusion

Nurses in the Arab ethnic minority demonstrate lower self-assessed nutrition and family eating and activity habits compared to non-health professionals. In addition, being part of the nursing profession, higher BMI, low self-assessed nutrition, and low food literacy were found to be associated with low family eating and activity habits among the Arab ethnic minority in Israel. These health risk behaviors are aligned with other socioeconomic risks experienced by the Arab ethnic minority. The results of the current study offer an additional explanation for the elevated prevalence of obesity and chronic non-communicable diseases within the Arab ethnic minority population in Israel.

The current study highlights the paradox whereby, while healthcare professionals are expected to model healthy behaviors, they often struggle to maintain them in their own lives. By acknowledging the impact of nurses’ health behaviors on their families and implementing targeted interventions, health organizations and policymakers can facilitate the adoption and maintenance of healthier lifestyles among nurses. This initiative may yield benefits for the nurses, patients, and communities they serve, particularly among minorities. Considering the demanding nature of the nursing profession and nurses’ essential role in promoting public health and preventive medicine, prioritizing their well-being is a critical investment in a healthier future.

## Abbreviations

BMI: Body mass index
*SFLQ*: Short food literacy questionnaire
SANS: Self-assessment nutrition score
*FEAHQ*: Family eating and activity habits questionnaire

## Declarations

### Data availability statements

The data that support the findings of this study are not openly available due to reasons of sensitivity and are available from the corresponding author upon reasonable request. Data are located in controlled access data storage at the University.
